# An Integrated Model of Patient and Staff Satisfaction Using Queuing Theory

**DOI:** 10.1109/JTEHM.2015.2400436

**Published:** 2015-02-06

**Authors:** Alexander Komashie, Ali Mousavi, P. John Clarkson, Terry Young

**Affiliations:** Wycliffe HallOxford UniversityOxfordOX2 6PWU.K.; Engineering Design CentreDepartment of EngineeringUniversity of CambridgeCambridgeCB2 1PZU.K.; Systems Engineering Research GroupBrunel UniversityUxbridgeUB8 3PHU.K.; Department of Computer Science and MathematicsBrunel UniversityUxbridgeUB8 3PHU.K.

**Keywords:** Patient satisfaction, staff satisfaction, queuing theory, waiting time, service time

## Abstract

This paper investigates the connection between patient satisfaction, waiting time, staff satisfaction, and service time. It uses a variety of models to enable improvement against experiential and operational health service goals. Patient satisfaction levels are estimated using a model based on waiting (waiting times). Staff satisfaction levels are estimated using a model based on the time spent with patients (service time). An integrated model of patient and staff satisfaction, the effective satisfaction level model, is then proposed (using queuing theory). This links patient satisfaction, waiting time, staff satisfaction, and service time, connecting two important concepts, namely, experience and efficiency in care delivery and leading to a more holistic approach in designing and managing health services. The proposed model will enable healthcare systems analysts to objectively and directly relate elements of service quality to capacity planning. Moreover, as an instrument used jointly by healthcare commissioners and providers, it affords the prospect of better resource allocation.

## Graphical abstract

1. Healthcare provision may be considered as involving the patient side of care and the staff side of care. 2. Analytical models exist for modelling patient or customer satisfaction. 3. We develop an analytical model for understanding staff satisfaction. 4. We propose an integrated model of patient and staff satisfaction by superimposing the staff satisfaction on top of patient satisfaction to determine the Effective Satisfaction Level (ESL) using Queuing Theory.

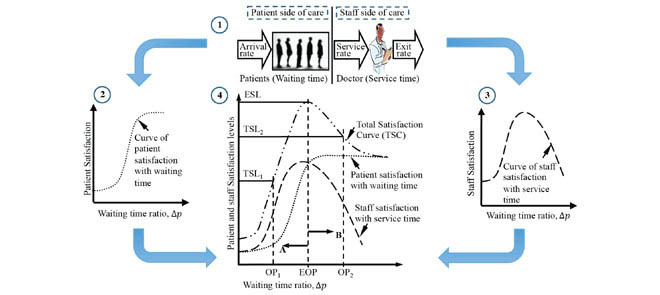

## I. Introduction

Patients’ satisfaction is generally accepted as a key indicator of the quality of care [1]–[3]. Patients’ waiting time is also considered as one measure of access to healthcare [4], [5]. In Great Britain and other developed countries, the goal in recent years has been to focus on improvements in patient experience often through setting challenging targets for healthcare providers [6], [7]. This practice of setting targets for healthcare providers especially in the UK has led to some improvement in care but also some unexpected practices on the part of providers that result in bad experiences for the patient [8], [9]. For instance, several inappropriate practices by National Health Service (NHS) Trusts in England as a result of performance targets have been found. These include, staff making changes to the records of thousands of patients in other to meet the target, ambulance Trusts were found to have corrected response times in order to meet Government target of 8 minutes and in some cases patients are held in ambulances outside Accident and Emergency departments until staff are confident of meeting the four hour target [8]–[12]. This evidence provided the main motivation for the current work. The starting question was this – “why will healthcare professionals trained to provide the best care for patients engage in practices that are harmful to the patients?” From the perspective of queuing theory, and by the consideration of Little’s Law [13], the authors identified three key variables of the problem that are very closely linked - Waiting time, Service time (or service rate) and Number in system. If these are so closely linked, then it may be expected that setting targets for waiting time - the patient side of care, may have indirect consequences for service rate – on the staff side of care - if nothing is done about the number in system. The authors, therefore, reasoned that the ideal of shaping healthcare provision around patients’ needs and preferences [14] must recognize the demand this places on staff resources [15] and the risk to quality when staff are stressed [16]–[18]. This led to the particular interest in service time (or the time staff spend with patients) in the current paper. A recent study has shown that in some situations much needed care is left undone due to staff not having enough time for tasks [15]. This recognition creates a triplet of challenges to those in service provision, namely to optimize patient satisfaction, staff satisfaction and service efficiency. The need to understand the interaction between patients’ needs, staff needs and service efficiency (or service quality) has been recognized for many decades. For instance, Donabedian [19] in his 1966 seminal paper on healthcare quality underscored this need by stating that *“…before one can make judgments about quality, one needs to understand how patients and physicians interact and how physicians function in the process of providing care.”* More recently, Oliva & Sterman [20] in a study in the service sector found that employees will reduce the time spent with customers and/or work longer hours to meet throughput targets. Furthermore, they noticed that in the absence of an understanding of the interaction between customers, staff and quality, management often interprets this reduction in time spent with customers as a productivity gain, leading to even tighter performance targets and eventually an unintended *“erosion of service quality”*. The challenge of understanding this interaction between patients’ needs, staff needs and service efficiency is the motivation for this paper.

To address this challenge, the paper reports on an approach that adds new methods to established ways of modelling healthcare processes. The contribution of the paper lies both in the integrated solution (Effective Satisfaction Level (ESL) model) proposed and the staff satisfaction model developed. This paper attempts to introduce the concept of Queuing Theory into the challenge presented above. This focuses the problem on patients’ satisfaction with waiting time and staff satisfaction with service time.

The paper first presents previous research involving traditional applications of Queuing Theory in Healthcare and customer satisfaction research with its application to patient satisfaction with waiting time. Secondly, the paper argues that despite significant work on staff satisfaction, no model exists that explains staff satisfaction behavior with service time and reports an empirical work that fills this gap. Thirdly, the proposed Effective Satisfaction Level (ESL) model, believed to be the best trade-off between patients and staff needs, is presented before finally finishing with some discussion and conclusions.

Some terms are used in the paper which are important for understanding the concepts presented. These terms are defined as follows:

• *Waiting Time Ratio:* The ratio of the difference between expected waiting time and actual waiting time to expected waiting time, $\Delta _{p}$ (subscript $p$ relates to patients).
• *Service Time Ratio:* The ratio of the difference between actual service time and ideal service time to the ideal service time, $\Delta _{s}$ (subscript $s$ relates to staff).
• *Satisfaction Level:* The estimated value of the satisfaction of patients or staff based on their expected waiting times or ideal service times.
• *Operating Point (OP):* A specific value of actual service time or its corresponding actual waiting time, with corresponding values of $\Delta _{s}$ and $\Delta _{p}$.
• *Total Satisfaction Curve (TSC):* The graph of the weighted sum of patient satisfaction and staff satisfaction levels at a specific value of expected waiting time and ideal service time.
• *Total Satisfaction Level (TSL):* A value of satisfaction on the TSC.
• *Effective Satisfaction Level (ESL):* The maximum TSL on the optimum TSC.
• *Effective Operating Point (EOP):* The operating point that corresponds to the ESL.

## II. Previous Research

### A. Queuing Theory: Relationship Between Service Time and Waiting Time

The field of queuing theory originated in the early 1900s and is well established with applications in diverse areas including manufacturing, computing, telecommunication and healthcare [21]. Within healthcare, bed occupancy has received significant attention with two examples being cooper & Corcoran [22] and Gorunescu et al. [23]. Several researchers, however, have undertaken to survey the breadth of the application of queuing theory in healthcare [24]–[26]. Of interest to the current study is the finding of a more recent survey by Fomundam & Herrmann [27]. The researchers found a diversity of application of queuing theory in healthcare including waiting time and utilization analysis, system design, appointment systems and system sizing. They, however, concluded that performance targets imposed on healthcare services are only likely to lead to congestions and poor quality of service and are unlikely to be a successful approach to containing and reducing healthcare costs. This finding and those of Ball et al. [15] of valuable care being left undone by nurses on wards make the need to better understand the symbiotic relationship between the patient side of care and the staff side of care urgent. This paper seeks to provide one way of looking at this problem.

**FIGURE 1.**
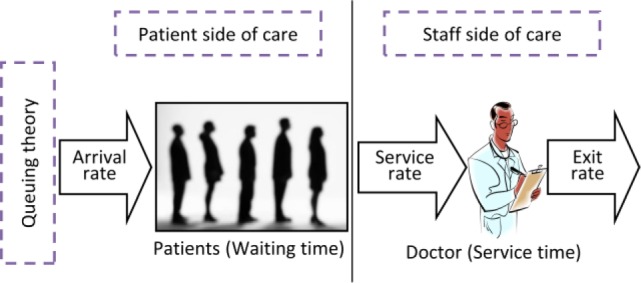
An illustration of a basic healthcare process showing a single doctor serving a single queue of patients.

**FIGURE 2.**
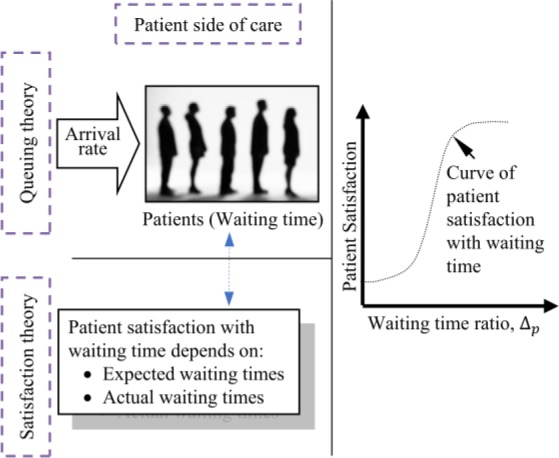
Behavior of patient satisfaction with waiting time.

**FIGURE 3.**
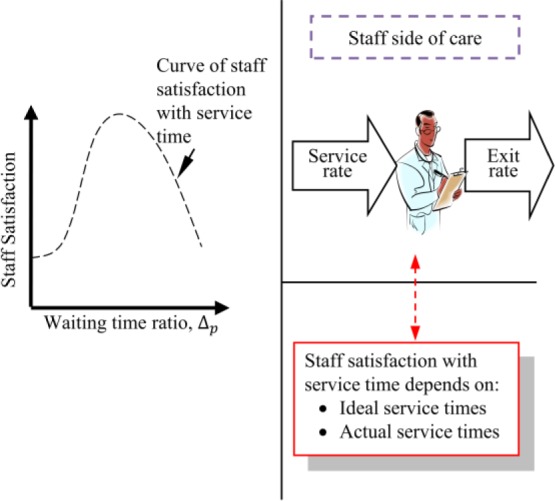
Behavior of staff satisfaction with service time.

**FIGURE 6.**
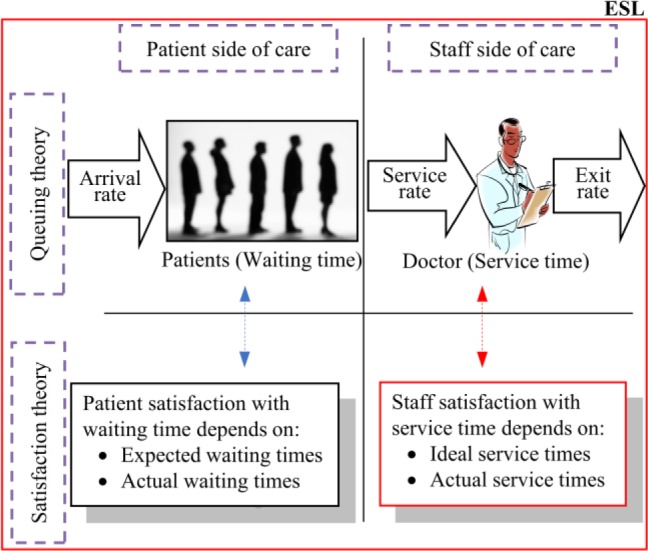
Conceptual representation of the ESL concept.

**FIGURE 7.**
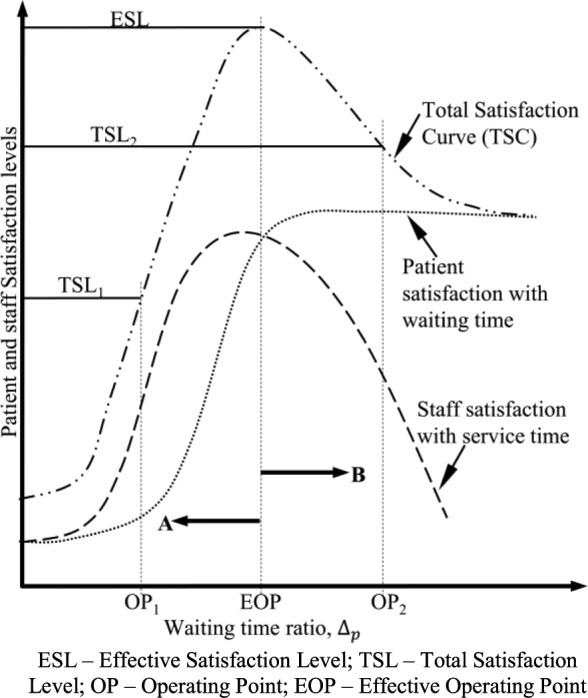
Hypothetical illustration of the effective satisfaction Level (ESL).

Figure 1 is an illustration of a simple healthcare delivery process with a single server (a doctor) and a single queue with patients. This and figures 2, 3, 6 and 7 were developed by the authors to help present the concepts in this paper in a clear and logical manner. The discussions in the rest of this paper will be developed around this basic illustration of the healthcare process.

The figure distinguishes the patient side of care from the staff side of care and indicates that Queuing Theory provides well established methods for understanding these two sides in terms of the relationship between waiting time and service time [28]. A summary of this relationship for a basic queuing discipline employed in this paper is presented in appendix A.

Focusing on the patient side of care, the paper will next provide a summary of previous work into measuring patient satisfaction and its application to waiting time.

### B. Measuring Patients’ Satisfaction Levels

Satisfaction research in healthcare has focused predominantly on patients [29]–[33], although there is background in customer and employee satisfaction [34]–[36]. The most popular method has been through surveys of patients. Analytically, the expectancy disconfirmation model [37], first proposed by Anderson [38] and confirmed by Oliver [39] is well attested and, critically, provides a useful connection to waiting and service times. Anderson notes that the level of satisfaction or dissatisfaction depends upon the users’ expectations of what they will receive and their perception of what they have received. For a review, see [40].

Meanwhile, Kahneman & Tversky [41] with their Prospect Theory, argue that people generally determine value by the changes in wealth or wellbeing, rather than the final states. This idea, combined with the expectancy disconfirmation model [39], has led to the development of analytical models for calculating satisfaction [42] and a better understanding of the behavior of patient satisfaction with waiting time as shown in figure 2. The curve shown in figure 2 is often approximated to a hyperbolic tangent function with a number of modified parameters for modelling purposes as shown in Mousavi et al. [42].

It is important to note that this paper focuses on patient satisfaction with waiting because waiting time is one of the key factors in care delivery but not the only one. It is also known that waiting time is one of the measures of access to healthcare [4], [5]. It is hoped that further research on the subject will lead to models that incorporate multiple factors.

## III. Measuring Healthcare Staff Satisfaction Levels

Unlike the patient side of care, the authors could not find any analytical model in the literature for calculating the satisfaction of staff with service time. It is not claimed here that service time, considered the time staff spend with patients, is the only determinant of their satisfaction. It is known that factors such as supervisory support, pay, development opportunities, work environment and many more also affect staff job satisfaction in healthcare [43], [44]. The choice of service time, was influenced by the observations of the effects of waiting time targets that motivated the current research. Several inappropriate practices have been found in the English health service that included leaving patient waiting in ambulances until staff were confident of meeting waiting time targets [8]–[12]. The empirical research was therefore designed around investigating and modelling the nature of staff satisfaction with service time. The authors acknowledge that a simpler model could have been developed in terms of the utilization factor, which is the ratio of arrival rate to service rate for a single server system [28], but admit that the current form of the model is constrained by the initial design of the research. Furthermore, the authors have learnt from experience working in healthcare that most healthcare practitioners will not be familiar with the concept of the utilization factor but are more likely to understand service time as the time they spend with patients. This is also supported by our data collection process in which we interviewed 68 doctors and nurses in two Accident and Emergency departments and found they were pleased to communicate in terms of service time. As a starting point, a hypothetical behavior was considered as shown in figure 3. The goal here is to connect staff satisfaction with service time through an empirical study and thus to fill a gap in the literature.

The study began by selecting a double hyperbolic tangent function, similar to that already proposed for customer satisfaction [42] and fitted empirical staff satisfaction findings to it. The double hyperbolic tangent function was employed rather than a single hyperbolic tangent function because it was hypothesized, as in figure 3, that staff satisfaction would decline if staff were rushed, but also if they were impeded in the timely discharge of their duties. The data collection methods and model development are discussed in the following section.

### A. Data Collection and Model Development

**FIGURE 4.**
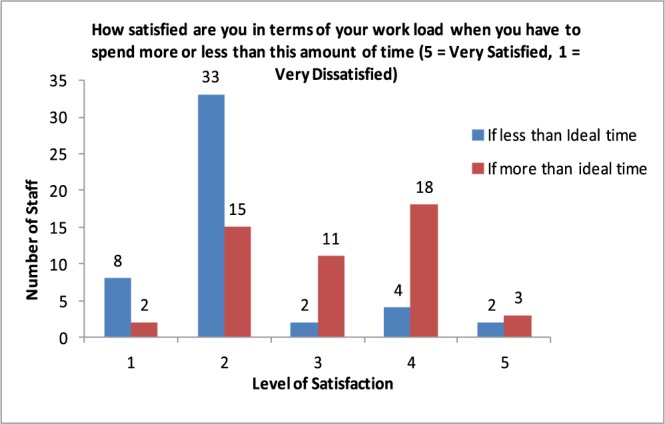
Summary of data from staff satisfaction interviews showing satisfaction with service time less or more than ideal service time.

At the time of this research, a major evaluation project around Accident and Emergency involving a satisfaction survey was already underway, using a questionnaire with a five point Likert scale. A subset of these questions were initially used but this only showed how satisfaction rose or fell with longer or shorter service times. Figure 4 summarizes the empirical data from the semi-structured interview with 68 doctors and nurses in two Accident and Emergency departments in London. It can be seen that the majority of staff are either dissatisfied or very dissatisfied when they spend less time with patients than what they thought was ideal. A significant number also were neutral, dissatisfied or very dissatisfied if they spent more than the ideal time.

**FIGURE 5.**
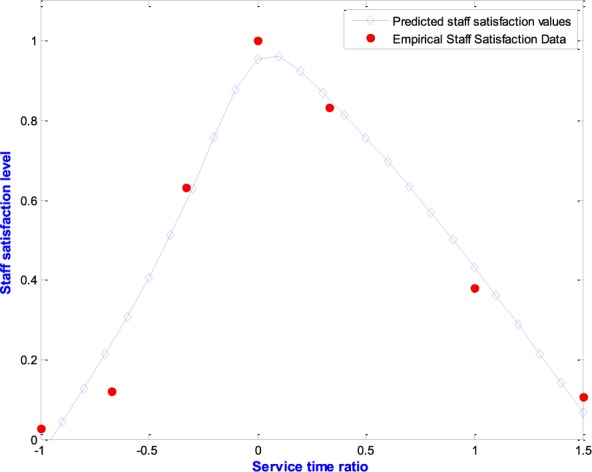
Behavior of staff satisfaction with service time based on empirical data.

Staff were further interviewed in order to probe their satisfaction with service time in more detail. In each interview a service time norm was established for three stages of the Accident and Emergency (A&E) pathway (triage, first assessment, treatment) and staff members were then asked how their sense of satisfaction changed at five, fifteen and twenty minutes away from that norm on either side. This data is the basis for the staff satisfaction behavior model shown in figure 5. In all, 68 doctors and nurses in two accident and emergency departments were interviewed.

The shape of the raw data in figure 5 led to a search for a model that has a bell-like shape. A number of standard models were considered including the inverse catenary, inverse $2^{\mathrm {nd}}$ order polynomial and the double hyperbolic tangent function. As shown in figure 5 and equation 1, The idea of the double hyperbolic tangent function (see appendix B) was inspired by the use of the single hyperbolic tangent function to approximate the customer satisfaction curve as in Mousavi et al. [42].

The best-fit analysis on the empirical data was conducted using the MATLAB curve fitting toolbox and the result is as shown in figure 5 and the most representative model for staff satisfaction is given by equation 1

\begin{align} U\left ({ \Delta _{s} }\right )=& 0.25\tanh \left ({ 1.72\Delta _{s} }\right )\notag \\&+\,0.76\Delta _{s}\tanh {\left ({ -4.43\Delta _{s} }\right )+0.95} \end{align}

where, $\Delta _{s}$ is the service time ratio.

With this model it became possible to connect staff satisfaction with service time with patient satisfaction with waiting time and develop the concept of the Effective Satisfaction Level (ESL).

## IV. Determining the Effective Satisfaction Level (ESL)

Finally, the Queuing Theory relationship between waiting time and service time, patient satisfaction with waiting time and staff satisfaction with service time are integrated to develop the concept of the Effective Satisfaction Level (ESL). Figure 6 shows the various aspects put together.

The ESL is the maximum level of satisfaction on the optimum Total Satisfaction Curve (TSC) at a given value of ideal service time expressed in terms of $\Delta _{p}$, the waiting time ratio. The TSC is simply the plot of the weighted sum of the satisfaction of patients and staff at a given value of ideal service time and expected waiting time. In this paper, however, equal weighting is apply to the satisfaction of both patients and staff as we only focus on the development of the ESL concept at this stage. Figure 7 is a hypothetical representation of the ESL at one instance of the TSC. It must be noted that expressing the ESL in terms of the waiting time ratio ($\Delta _{p})$ has the effect of reversing the sense of the graph.

This is because $\Delta _{p}$ is calculated as the expected waiting time, minus actual waiting time, all divided by expected waiting time. The horizontal axis of figure 7, therefore, must be understood as representing increasing values of $\Delta _{p}$ but decreasing values of actual waiting time.

Operating at point “OP_1_”, in the direction of arrow “A” in Figure 7, (to the left of the EOP) means staff are taking longer than they expect for their processes and hence queues will increase and result in lower values of patient and staff satisfaction and therefore, a lower value of Total Satisfaction Level (TSL_1_). Similarly, operating at point “OP_2_”, in the direction of arrow “B” (to the right of the EOP) means staff are working faster than they would normally like to do. This will potentially reduce patient waiting time in the queue and therefore increase their satisfaction, but the decrease in staff satisfaction will result in a decrease in total satisfaction level to TSL_2_. It may be necessary at times to maximize patient satisfaction in this way but this may require a shared understanding in a staff team to be effective. In this case we may assume that the encounter is effective and leads to patient satisfaction, but this has not been included in our modelled.

### A. Experimental Results

**FIGURE 8.**
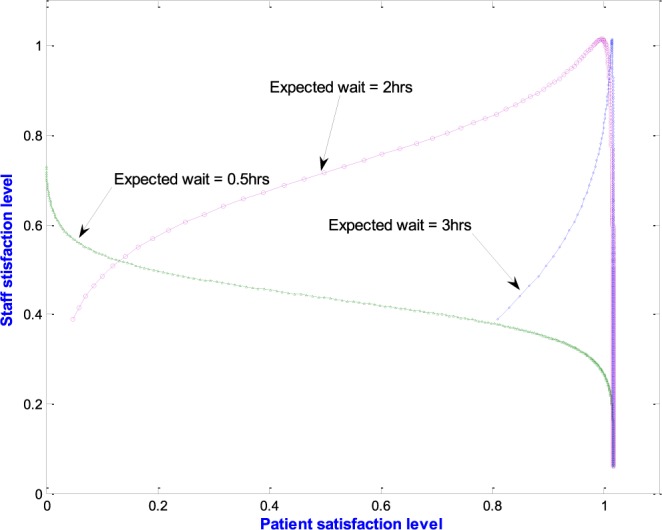
Variations in Patient Satisfaction with Staff Satisfaction.

The MATLAB software was used to implement the queuing models presented in equations 2 through 4 of appendix A together with the patient satisfaction model discussed in section IIB and the staff satisfaction model developed in section III. This allowed us to test the hypothetical behavior illustrated in figure 7 above and also the relationship between patient satisfaction with waiting time and staff satisfaction with service time. Figures 8 through 10 demonstrate how the Effective Satisfaction Level (ESL) may be found.

Figure 8 shows one result from the implementation which reveals how patient satisfaction with waiting time varies with staff satisfaction with service time. The figure shows three curves for which the expected patients’ waiting times are 0.5hrs, 2hrs and 3hrs respectively. In each case the ideal service time is 2hrs. As expected, if the patient expectation is 0.5hrs, there is no way to satisfy both patients and staff. However, if patients expect 2hrs, then there is a solution where both patients and staff are close to maximum. The reason the curve does not quite go through the point (1, 1) is because the model rewards shorter-than-expected delays with slightly higher levels of patient satisfaction than that corresponding to the expected delay. A patient would be happy to leave after two hours, but a little happier to leave before that. This becomes clear if patients expect to wait 3hrs and get through in 2.

**FIGURE 9.**
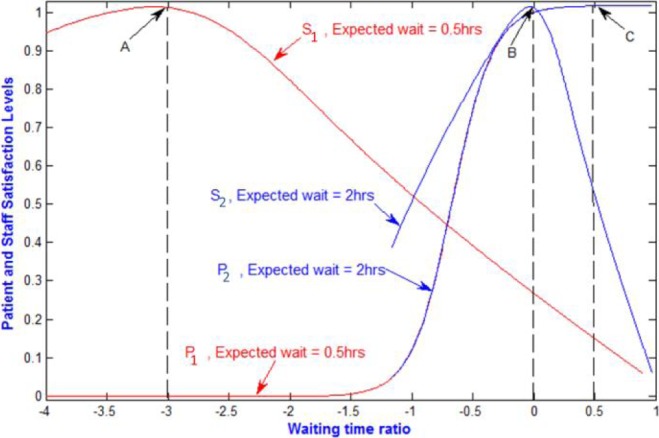
Plot of patient and staff satisfaction against waiting time ratio.

Figure 9 shows a plot of patient and staff satisfaction curves against the waiting time ratio, $\Delta _{p}$, (as in figure 7). However, this is based on model output. The figure shows two pairs of curves (S_1_, P_1_ and S_2_, P_2_ for the expected waiting times of 0.5hrs and 2hrs respectively. Point A is where maximum staff satisfaction occurs on S_1_ and point C is where maximum patient satisfaction occurs on P_1_. These two points are far apart, which means that when a target of 30 minutes is imposed on waiting times, maximum staff satisfaction or maximum patient satisfaction will result in the dissatisfaction of the other.

Similarly, S_2_ and P_2_ are the staff and patient satisfaction curves at an expected waiting time of 2hrs. It is observed that in this case, maximum staff satisfaction occurs at point B which is much closer to the maximum patient satisfaction than point A. Also the difference between staff satisfaction and patient satisfaction levels is almost negligible. Note that ideal service time is fixed at 2hrs. The ESL (see figure 7) may, therefore, be expected to occur in the vicinity of point B, where staff satisfaction and patient satisfaction are at or near their maximum. This shows that the model has the potential to accurately predict what may be expected from doctors and nurses for a desired level of patient experience.

It is suggested that the ESL must be the goal of most healthcare systems. Even when capacity constraints make it difficult for a system to operate at the ESL, it may still be desirable to know how far the system is from its ESL. This concept uniquely provides a meaningful method for assessing the capability of a healthcare system to examine the validity of any arbitrary target.

The introduction of the ESL provides numerous opportunities for exploring and better understanding the queuing problem in a healthcare system. Examples of questions that may be explored are: 1. What is the optimum level of resources required for a system to operate at the ESL? 2. What level of demand can a system accept without moving off the ESL? Or, 3. What is an acceptable number of patients waiting in a queue at the ESL?

**FIGURE 10.**
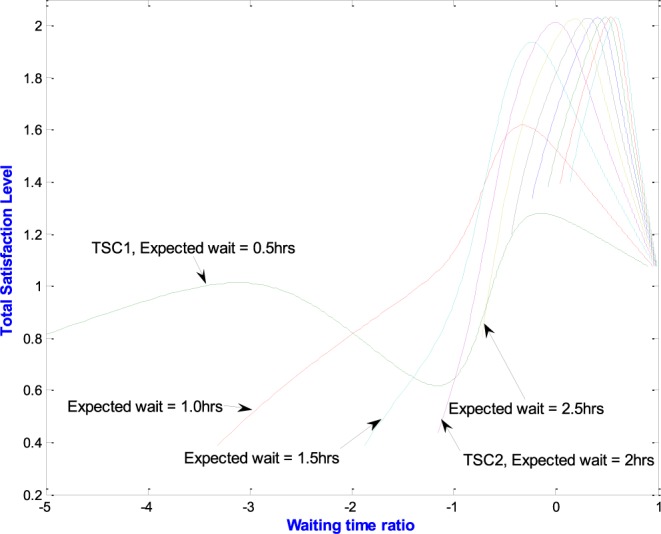
Variations in total satisfaction curves (TSC).

Figure 10 further shows the Total Satisfaction Curve (TSC) by which the ESL is identified. TSC_1_ is the resulting total satisfaction curve when the expected waiting time is 0.5hrs whilst TSC_2_ is the total satisfaction curve when the expected waiting time is 2.0hrs. Other TSCs are shown for various values of expected waiting time. The curves show that the maximum value of the total satisfaction of staff and patient is higher when the expected waiting time is 2.0hrs than when it is 0.5 hrs. This point of maximum total satisfaction, therefore, corresponds to high values of both staff satisfaction and patient satisfaction and is what we call the Effective Satisfaction Level (ESL).

## V. Discussion and Conclusions

It has been found that the approach to managing queues in a healthcare system through arbitrary targets and performance ratings is problematic [8], [10]. The authors argue that in the management of queues in the British National Health Service, significant emphasis is placed on satisfying patients without understanding the implications on the staff that are the key resources in the system. This paper has focused on showing the link that exists between the service time of staff and the waiting time of patients and hence between the satisfaction of staff with service time and patient satisfaction with waiting time.

Based on empirical data, a model for estimating the satisfaction of staff with service time was developed. The development of this model made it possible to directly relate the satisfaction of staff to that of patients. The Effective Satisfaction Level (ESL) was developed by analytically relating the satisfaction of staff to that of patients. Through the concept of the ESL we present an argument that a synergy between patient satisfaction and staff satisfaction is the key to sustainable improvement in healthcare quality because it is a more transparent approach. This paper, therefore, suggests that most healthcare systems must ideally operate at the ESL and where this is not possible due to resource constraints, it is still important to know how far a system is from its ESL. The results have shown that the ESL occurs when the ideal service time is close to or equal to the actual service time with the corresponding actual waiting time also close to or equal to the expected waiting time. This means that there is the potential to be able to accurately predict what may be expected from doctors and nurses for a desired level of patient experience.

### A. Assumptions, Limitations and Further Work

A number of assumptions were made in the application of queuing theory in this paper:

1. The entire A&E system has been considered as a single server and a single queue (M/G/1) system [28]. As a future work, a more complex (M/G/n) will be studied.
2. It was also assumed that the time between patient inter-arrival times into the A&E system is random and exponentially distributed.
3. The service times were also assumed to be random and follow any general distribution.
4. Patients in queue were also assumed to be served on a First Come First Served (FCFS) basis.
5. For simplicity, other factors such as communication, cleanliness, dignity and access to care which may influence satisfaction, were not included at present.

A number of limitations may be identified with regard to this study which also provide opportunities for further work. Firstly, one may argue that the satisfaction of patients is not just about waiting time. Whilst we admit the truth of this argument, we believe that most of the factors that affect patient experience and eventually the quality of care such as communication, confidence in staff, dignity, access to care and cleanliness may also be time related [15]. Hence a study of the relationships of such factors with staff service times planned as the future direction of this research may facilitate the inclusion of multiple factors and eventually the development of a more unifying satisfaction model.

Secondly, there are also the realities of multiple visits and multi-purpose visits which are currently beyond the scope of this model, however, some possibilities for extending or applying the current model may be considered. For instance if the idea of multiple visits is considered the case where a patient visits multiple servers or stations (e.g. receptionist, nurse, doctor, laboratory), then this becomes the problem of a network of queues with the possibility of repeated visits to some stations and exogenous and endogenous arrivals to any station [28], [45]. The simplest analytical model for such a network of queues is formulated by considering each node in the network as a station analogous to, and treated as, a single server system [45]. For such a network, one way of using the current model may be to apply it in its current form to each individual station in order to obtain actual waiting times at each station and to derive the overall waiting time for the network as a linear combination of these. The authors are aware of the challenges involved in solving complex analytical problems of queuing networks and in particular the fact that the $M/G/k$ model which is the direct extension of the type employed in this work remains essentially an open problem [46]. We also acknowledge that, analytically, the use of the utilization factor, *rho* may allow ease of manipulation but can foresee some practical challenges as *rho* is not directly measurable. It is, however, of interest to the authors to explore the use of *rho* in future extensions of the model. Another way the model may be used is to implement it in a Discrete Event Simulation (DES) model which is an alternative to an analytical solution.

Thirdly, the assumptions listed above show that the mathematical formulation is not a perfect representation of a real A&E system. However, our goal at this stage is to provide a quantitative explanation to a seemingly obvious phenomenon. The importance of this approach is that it enables us to more easily control the parameters that influence the phenomenon or the interaction between staff and patients. This concept, as with every scientific theory, requires further research and enhancement. We consider that an exploration of the extension of this concept to a service system as a network of queues is a logical next step.

### B. Implications

The understanding of the relationship between the satisfaction of staff and that of patients will lead to more realistic expectations of healthcare systems and their performance or non-performance. It should be possible to know the extent to which increases in the satisfaction of patients by the reduction of waiting times may be pursued in a system of limited resources.

There should also be no more need for healthcare managers to employ “coping” methods [8], [10], [47], since any unrealistic expectations should be detected by the proposed model.

It is finally emphasized that a healthcare system may ideally operate at the ESL and where this is not possible due to resource constraints, it is still important to know how far a system is from its ESL and how much resource is required to move it there.

## Appendix A Queuing Theory Relationship Between Waiting Time and Service Time

Queuing theory is a well-established field of research that has been applied to many systems including healthcare. Several queuing models exist, from single stations to complex networks of queues implementing several queuing disciplines. In this paper we employ one of the most basic queuing models – *M/G/1* in order to demonstrate how patient satisfaction with waiting time may be connected to staff satisfaction with service time. These models often involve a set of assumptions, known parameters, unknown parameters and analytical solutions to some of the standard models as briefly discussed below.

### A. The Initial System Assumptions

We assume a healthcare system with only one doctor/nurse serving a single queue of patients. The queuing discipline is First Come First Serviced (FCFS) - this may be extended to cover a priority based queues. The arrival process is a Poisson distribution therefore inter-arrival times are exponentially distributed [48]. Service time is any general distribution (triangular in this case).

### B. Known Parameters

The following parameters are known for the above system;

Average inter-arrival rate $= \lambda $

Average service rate $= \mu $

Average service time $= S = \mu ^{-1}$

Number of servers = 1

Utilization factor $= \rho = \frac {\lambda }{c\mu }$

### C. Unknown Parameters

It is desired to know: The average total or throughput time for patients in the system, $W_{T}$; The average time in queue, $W_{q}$; Total Number of patients in the system or work-in-progress, $N_{p}$.

According to Little’s Law [13], which is the basis of the following analysis, there exists a definite relationship between work-in-process, production rate and throughput time. The statement of the law is as follows:

\begin{equation*} N_{p} = \mu W_{T} \end{equation*}

Where,

$N_{p} =$ Number of patients in process (WIP)
$\mu =$ Servicing rate of patients (Production rate)
$W_{T} =$ Average total time spent by a patient (Throughput time)

### D. M/G/1, FCFS (Exponential Inter-Arrival, any General Service Distribution, 1 Server, With a First Come First Served Queuexs)

The M/G/1 model is a standard model in the field of queuing theory for which a standard set of solutions exist [28], [45]. The following standard solutions were employed in this study:

Estimated total time of patients,

\begin{equation*} E\left ({ W_{T} }\right )=E\left ({ S_{act} }\right )+ \frac {\lambda E\left ({ S_{act}^{2} }\right )}{2\left ({ 1-\rho }\right )} \end{equation*}

where $E\left ({ S_{act} }\right )$ is the expected value of the actual service time of staff.

Also, estimated total number of patients in system,

\begin{equation*} E\left ({ N_{p} }\right )= \rho + \frac {\lambda ^{2}E\left ({ S_{act}^{2} }\right )}{2\left ({ 1-\rho }\right )} \end{equation*}

therefore, the expect value of the actual waiting time may be simply determined as:

\begin{equation*} E\left ({ W_{act} }\right )=E\left ({ W_{T} }\right )-E\left ({ S_{act} }\right ) \end{equation*}

The expected or ideal service time for staff, $S_{ideal}$, has been determined from interviews with staff for three of the Accident and Emergency (A&E) process stages. Based on these definite relationships, we argue that when a target is imposed on the system as to how long patients should wait in a queue, $W_{act}$, this can then be translated into the actual or required service time for staff, $S_{act}$, using equations 3 to 5, which then helps to determine the satisfaction of staff with service time at that target.

This attempt to connect patients’ waiting time to staff service time allows the idea of actual waiting time to be conceptualized in a number of ways. For instance, it may be possible to imagine an ideal waiting time, $W^{i}_{act}$, which is the best possible level of service (best possible waiting time) that the system can provide within given resource constraints. Also, it may be of interest to consider the prevailing level of operating performance, $W^{o}_{act}$ and compare this to the best possible performance. Using $W^{i}_{act}$ to estimate the satisfaction of patient and staff may not give a good reflection of reality. This will, however, be very useful in determining how well the system is performing against what it is capable of doing.

$W^{o}_{act}$ on the other hand is the current level of performance which may be easily estimated from operating conditions and should give a better reflection of satisfaction values.

## Appendix B Staff Satisfactioin Model

In order to be able to connect patient satisfaction with waiting time with staff satisfaction with service time, it was necessary to develop an analytical model of the behavior of staff satisfaction with service time since there was nothing of the kind in the literature.

Mathematically, we started by formulating the staff satisfaction model as a double hyperbolic function following the concept of customer satisfaction in Mousavi et al (2001) as follows;

\begin{align} U\left ({ \Delta _{s} }\right )=&\omega _{1}\tanh \left ({ \beta _{1}\Delta _{s}+\lambda _{1} }\right )+\gamma _{1}\notag \\&+\,\omega _{2}\Delta _{s}\tanh \left ({ \beta _{2}\Delta _{s}+\lambda _{2} }\right )+\gamma _{2} \end{align}

where

$\omega _{1}$ is the range factor for the first half of the curve, $\omega _{1}\ne 0$

$\beta _{1}$ is the sensitivity factor for the first half of the curve, $\beta _{1}\ne 0$

$\lambda _{1}$ is the horizontal location factor for the first half of the curve

$\gamma _{1}$ is the vertical location factor for the first half of the curve

$\omega _{2}$ is the range factor for the second half of the curve, $\omega _{2}\ne 0$

$\beta _{2}$ is the sensitivity factor for the second half of the curve, $\beta _{2}\ne 0$

$\lambda _{2}$ is the horizontal location factor for the second half of the curve

$\gamma _{2}$ is the vertical location factor for the second half of the curve and

\begin{equation*} \Delta _{s}=\frac {S_{act}-S_{ideal}}{S_{ideal}} \end{equation*}

$S_{ideal}$ is the ideal service time, thus time staff are happy to spend with patients to enable them to work effectively.

$S_{act}$ is the actual service time, that is the amount of time the staff actually spend.

The empirical data collected by interviewing 68 doctors and nurses in two Accident and Emergency departments in London was fitted to this model in order to determine the value of the constants. The result has been discussed in the paper in section III.
